# Understanding Fetal Alcohol Spectrum Disorders (FASDs): Toward Identification of a Behavioral Phenotype

**DOI:** 10.1100/tsw.2008.75

**Published:** 2008-09-21

**Authors:** Kelly Nash, Erin Sheard, Joanne Rovet, Gideon Koren

**Affiliations:** The Hospital for Sick Children, Toronto and the Ontario Institute for Studies in Education, University of Toronto, Toronto, Canada

**Keywords:** fetal alcohol spectrum disorder, developmental neurocognition, behavioral phenotype

## Abstract

Fetal alcohol spectrum disorders (FASDs) currently represent the leading cause of mental retardation in North America, ahead of Down syndrome and cerebral palsy. The damaging effects of alcohol on the developing brain have a cascading impact on the social and neurocognitive profiles of affected individuals. Researchers investigating the profiles of children with FASDs have found impairments in learning and memory, executive functioning, and language, as well as hyperactivity, impulsivity, poor communication skills, difficulties with social and moral reasoning, and psychopathology. The primary goal of this review paper is to examine current issues pertaining to the identification of a behavioral phenotype in FASDs, as well as to address related screening and diagnostic concerns. We conclude that future research initiatives comparing children with FASDs to nonalcohol-exposed children with similar cognitive and socioemotional profiles should aid in uncovering the unique behavioral phenotype for FASDs.

## INTRODUCTION

After decades of research, the damaging effects of prenatal alcohol exposure have been well documented. Historically, suggestions that prenatal alcohol exposure (PAE) may lead to neurodevelopmental impairments were dismissed for explanations en vogue at the time, such as heredity, leaving the teratogenic effects of PAE unrecognized until the late 1960s. At that time, consistent results emerged in France and the U.S.[1], drawing similar conclusions regarding the appearance and behavior of alcohol-exposed offspring. By 1973, the constellation of symptoms resulting from PAE was named fetal alcohol syndrome (FAS)[2]. It is now widely recognized that PAE may lead to fetal alcohol spectrum disorders (FASDs), a term that encompasses both full-blown FAS, as well as fetal alcohol effects (FAE) or alcohol-related neurodevelopment disorder (ARND), which present as behavioral or cognitive abnormalities in the absence of dysmorphology[3]. Even at low exposure levels, PAE has been associated with long-term cognitive difficulties[4] and there is no known safe ingestion limit[5]. To date, researchers are still working to understand fully the scope and mechanisms of prenatal alcohol effects.

Early research appeared to indicate that PAE represented a continuum of severity of effects, with FAS being the most severe form[6]. However, recent research has demonstrated that this is not the case; individuals with FAS, partial FAS, and ARND exhibit equivalent impairments[7] and qualitatively similar cognitive profiles[8,9]. Although the pattern of primary disabilities is similar in FAS and ARND[7,10], ARND may, in fact, be even more susceptible to negative long-term outcomes than FAS[11]. Due to the presence of characteristic facial dysmorphology, a child with FAS is more likely to be identified and diagnosed early in life, while diagnosis is often delayed or missed in ARND, even though the deficits are evident by 4 years of age or earlier[11]. Despite years of research highlighting the negative impact of PAE, it is still a leading cause of mental retardation[12] and neurobehavioral deficits in cognitive and socioemotional functioning[13]. In the U.S., the prevalence of FAS is estimated at 0.5–2/1000 and FASDs at 10/1000 births[14], and comparable estimates of 1–6/1000 live births have been made in Canada[6], with variations within and between countries[15]. Despite a number of primary prevention efforts, the rates have remained unchanged over the years.

## Screening and Diagnosis

The clinical diagnosis of FASDs is not simple. Rather, "FASDs" is an umbrella term for wide range of possible consequences of PAE. Our use of the term FASDs includes the full spectrum of disorders resulting from PAE. Diagnosis of FAS was first standardized by the Institute of Medicine[16] and is defined by International Classification of Disease (ICD-9 and ICD-10). Under IOM classification, four criteria must be met for a diagnosis of FAS: (1) severe intrauterine or postnatal growth deficiency with height and/or weight at less than 10th percentile; (2) craniofacial dysmorphology including smooth philtrum, thin vermilion, and small palpebral fissures; (3) central nervous system damage that is either structural or neurological; although functional impairment is also assessed, it is not sufficient for diagnosis; and (4) PAE, which may be confirmed, unknown, or disconfirmed. Diagnostic categories include FAS with or without confirmation of alcohol exposure, partial FAS, ARND, or alcohol-related birth defects (ARBD).

Alternative diagnostic methods include the University of Washington's 4-Digit Diagnostic Code[17], which ranks each of the four key features highlighted in the IMO guidelines on a Likert scale to derive a clinical code that differentiates FAS from other categories. The Washington Likert scales offer more precision in describing and measuring the features than the IOM guidelines (1 = absence of feature, 4 = extreme expression). Given the overlapping criteria, in practice, the IOM and Washington guidelines are often integrated in diagnosis. For instance, the Canadian Guidelines blend IOM and Washington Criteria in addition to criteria for diagnosing FAS, partial FAS, and ARND[6].

Given the time and resources required to diagnose FASDs, screening tools are being developed to identify at-risk individuals who require further assessment. Novel screening tools using biomarkers, such as fatty acid ethyl ester levels in meconium or hair, are currently being developed in an effort to support the more traditional diagnostic procedures[18]. Also promising are rapid and easy-to-administer screening tools. These screening tools may be especially helpful in order to access populations in remote or rural areas. One example screening tool, developed by Nash and colleagues[19], utilizes 12 items from a standardized tool for assessing behavior, the Child Behaviour Checklist, shown to differentiate reliably between FASDs, attention-deficit hyperactivity disorder (ADHD), and typically developing children. A specific subset of characteristics has been identified as unique to FASDs, which includes a lack of guilt, cruelty, acting young, lying or cheating, and stealing from home or stealing from outside of the home. In an effort to expedite the diagnostic process, children identified through screening could then be referred for diagnostic evaluation (see Table 1).

The importance of early diagnosis must be emphasized. Although the primary and secondary disabilities from PAE are extremely costly, debilitating, and lifelong, they can be mitigated with early intervention[11]. Current research findings show a substantial protective effect of early diagnosis and treatment in alleviating

**TABLE 1**
**Screening Checklist for FASDs Behavioral Phenotype**

*Step 1: Identifying behavior suggestive of FASDs*

The following questions should be asked of the child's parent/guardian to determine whether the child's behavior is suggestive of FASDs.

1. Does your child act too young for his/her age?
2. Does your child have difficulty concentrating, and can't pay attention for long?
3. Is your child disobedient at home?
4. Does your child lie or cheat?
5. Does your child lack guilt after misbehaving?
6. Does your child act impulsively and without thinking?
7. Does your child have difficulty sitting still/is restless/hyperactive?

If the parent/caregiver answers "yes" to at least six out of seven items, this is suggestive of FASDs with 86% sensitivity and 82% specificity.

If the child does not exhibit behavior consistent with ADHD (i.e., answer is negative for questions 2, 6, 7), then a score of three out of the four following questions needs to be positive:

1. Does your child lack guilt after misbehaving?
2. Does your child lie or cheat.
3. Is your child disobedient at home?
4. Does your child act too young for his/her age?

*Step 2: Differentiating FASDs from ADHD*

The child needs to exhibit two of the following three:

1. Does your child experience lack of guilt after misbehaving?
2. Does your child display acts of cruelty, bullying, or meanness to others?
3. Does your child act young for his/her age?

OR, the child needs to exhibit three of the following six:

1. Does your child experience a lack of guilt after misbehaving?
2. Does your child display acts of cruelty, bullying, or meanness to others?
3. Does your child act young for his/her age?
4. Does your child steal from home?
5. Does your child steal outside of home?
6. Does your child lie and cheat?

later-associated mental health problems[11]. Diagnosis of FAS may also be easier in young children because craniofacial dysmorphology becomes less pronounced over time[20]. The single biggest challenge to early intervention is delayed diagnosis.

## Cognitive Profile

An important question in FASDs research is whether or not PAE results in a clearly definable and identifiable cognitive-behavioral profile also known as a phenotype. FASDs certainly have a complex and varied phenotypical profile, although key cognitive and behavioral features are evident[13]. The resultant primary disabilities reflect the extensive and varied nature of the brain damage caused by PAE. Alcohol acts on the central nervous system through multiple mechanisms and impacts several cognitive domains. In contrast to the dysmorphology, the primary cognitive disabilities do not show age-related improvement[21] and persist into adulthood[11]. Although this is not an exhaustive list, primary disabilities in FASDs include reduced IQ, learning disabilities, lower achievement scores, language difficulties, and problems in attention, working memory, executive functioning, and socioemotional functioning.

A more thorough understanding of the cognitive-behavioral phenotype in FASDs is necessary for improving diagnosis, particularly as it relates to neuropsychological impairment and to developing effective interventions. Indeed, the severe consequences of PAE on the developing brain demonstrate the need for a diagnostic process that should more strongly emphasize the neuropsychological profile[6,9]. A comprehensive review of the cognitive deficits associated with FASDs is beyond the scope of this chapter; thus, we seek to highlight key areas of impairment and identify the gaps and limitations in our understanding of the cognitive-behavioral outcome in FASDs.

## IQ and Academic Achievement

PAE has a deleterious effect on IQ and academic achievement, and may result in profound learning disabilities. The earliest work on FASDs linked PAE to significant impairments in IQ, with the severity of dysmorphology relating to degree of mental deficiency[22]. Indeed, alcohol is widely recognized as the leading cause of preventable mental retardation, occurring more frequently than the other two most common birth defects combined[12] and the deficits endure across the lifespan[11].

Streissguth and colleagues[23] reported an almost 7-point decrease in IQ in 7-year-old children exposed to an ounce of alcohol per day mid-gestation. Severity of dysmorphogenesis correlates with IQ, with IQ scores ranging from 55 if manifestations are severe to a normal score of 82 with less pronounced manifestation[22]. Average IQ estimates using standardized instruments are typically between 65 and 70, although variability ranges from about 20 to 105 (normal). For instance, Abel[24] reported an average IQ of 67, with over half scoring below 70, indicating mental retardation. IQ is impaired on both the verbal and performance scales[25], although verbal deficits may become increasingly marked with age[26]. Unlike spelling and reading, a particular deficit in mathematics has been noted, with deficits exceeding those that would be predicted from IQ scores[4]. This impaired intellectual function contributes to academic difficulty[4], despite evidence that IQ is not the best predictor of academic achievement[27].

Children and adolescents with FASDs report negative academic experiences, and parents and teachers report below-average academic achievement. Average academic functioning falls at the grade 2–4 level[4], a level of functioning that does not solely reflect IQ and points to other contributing factors like a poor postnatal environment. Children with PAE are at increased risk for learning disabilities, and are more likely to be placed in remedial or special educational settings, to be suspended, or to drop out[28]. In children without full-blown FAS, however, the learning disabilities are sometimes mistakenly attributed to motivational issues rather than brain damage, compounding the problem[29] and limiting access to proper intervention. As the child ages and the academic demands increase, the problems are marked with greater task difficulty, a need for more monitoring, and an increasingly negative attitude toward academic work.

## Language and Communication

Language problems as indicated on IQ measures have been consistently noted; for example, Streissguth et al.[28] noted a discrepancy between verbal IQ and performance IQ, with greater impairment in the verbal realm. Indeed, language problems tend to present early in FASDs[30] and may be a trigger for referral. Initial language delays are often exacerbated hearing disorders, as hearing is important in normal language acquisition, reception, and expression[31]. In 3-year-old children, Greene and colleagues[32] found no relation between alcohol exposure and language in the absence of craniofacial dysmorphometry; however, some speech delay and language deficits may reflect a higher-level central auditory processing disorder[31]. Language delays are also evident into the school years, but because individuals with FASDs tend to have adequate social speech[33], the language and communication problems may go unnoticed or untreated. A child with FASDs may seem to be typically verbal, but there is often a striking contrast between their use of verbal language and comprehension and understanding.

The most salient impairments are in receptive language[27], resulting in difficulty understanding and interpreting information, whereas expressive language is stronger. That said, both components of language are impaired in FASDs, with 82% showing receptive and 76% showing expressive[34] performance greater than one standard deviation below the mean. The severity of language impairment in FASDs correlate with socioemotional disturbance[35], emphasizing the critical role of language in social cognition. Language is a significant area of impairment in FASDs, but there are few studies with a primary emphasis on language.

## Attention

Attentional deficits are a core problem in FASDs. However, the precise nature of these deficits, and more specifically the relation between attentional deficits in FASDs and ADHD, is not well understood. Linnet et al.[36] reviewed maternal factors in ADHD risk and found no significant relation between PAE and ADHD, although many others have found a link between PAE and ADHD symptoms[37], and ADHD prevalence may increase with amount of PAE[38].

ADHD is the most commonly reported comorbid disorder associated with FASDs[38], yet some researchers believe that the attentional profile in FASDs is unique or presents as an ADHD phenocopy. O'Malley and Nanson[37] reviewed literature on the FASDs-ADHD link and concluded that in FASDs, ADHD is more likely to be the earlier-onset inattentive subtype. Coles and colleagues[39] reported that children with FAS had more attentional problems in encoding and shifting focus, whereas nonexposed children with ADHD were most impaired in focused and sustained attention. Subtle differences in attentional profiles may help to differentiate ADHD-like symptoms resulting from PAE from ADHD. This point is particularly poignant given that some individuals with FASDs may be misdiagnosed as having ADHD as the primary diagnosis, resulting in missed or inappropriate interventions. Furthermore, the aspects of attention most impaired in FASDs – encoding and shifting – implicate a more general problem in working memory and executive functioning, respectively. Burden et al.[40] investigated the effect of PAE on the components of attention and found that working memory was the aspect of attention most adversely affected by PAE. Consistent with Coles et al.[39], no association between sustained or focused attention and PAE was found.

## Executive Functioning and Working Memory

Executive functioning (EF) refers to a broad spectrum of goal-oriented abilities that include working memory, planning, decision making, cognitive flexibility, inhibition, reasoning, and problem solving. Global deficits affecting both the verbal and nonverbal domains in multiple aspects of EF have been reported in FASDs from childhood to adulthood, independent of facial dysmorphology[9]. Although few studies have looked at the role of affect, emotional EF processing also appears to be impaired in FASDs[41]. Mattson et al.[42] demonstrate that EF impairments are not merely a reflection of component process deficits in basic skills such as reading or motor speed, but are true deficits in higher-level executive processes. Rasmussen[9] highlights the sweeping EF impairments as measured on a variety of tasks across the lifespan and independent of FASDs diagnosis. Deficits may be particularly evident in high demand settings such as school, as reflected by the fact that teacher ratings indicate greater EF difficulty than parent ratings[26].

Cognitive flexibility, for example, involves dividing and shifting attention. On a task requiring the child to meet changing criterion flexibly, as in the Wisconsin Card Sorting Task, children with FASDs will persevere on the wrong category after the criterion has shifted[39]. Planning is similarly impaired, illustrated by poor performance on tasks such as the Tower of Hanoi, which requires the participant to plan ahead to make the minimum number of moves necessary to replicate a pattern[43]. Deficits in inhibitory control have been reported and map onto reported behavioral problems of impulsivity, although inhibitory deficits may be task specific. For example, Mattson et al.[42] reported impairments in response-inhibition on a Stroop task requiring participants to inhibit one response (word naming) in lieu of the correct response (color naming), although this deficit has not been consistently found[43].

Working memory (WM) is a central feature of EF processes and is thought by some to be the core deficit in FASDs[43]. Deficits in the maintenance and manipulation of information are observed on measures such as forward and backward digit span[23], trigram recall with rehearsal prevention[44], and the n-Back task[45]. These WM deficits are thought to underlie the extensive EF deficits in FASDs[43] and may exceed impairment predicted by overall IQ([44], but see also [9] for deficits mediated by IQ). Although WM and EF deficits in FASDs are widely acknowledged, Rasmussen[9] highlights the paucity of interventions and treatments designed to target EF impairments; the undertaking of such important initiatives should serve to reduce the prevalence of secondary disabilities such as academic failure, sociobehavioral disturbance, and societal difficulty.

## Learning and Memory

In FASDs, learning and memory impairments are particularly evident in spatial memory and associative learning, and are primarily linked to acquisition rather than storage and retrieval of information[46]. The affected memory processes are mediated by the hippocampus and, not coincidentally, the hippocampus is thought to be particularly vulnerable to the effects of PAE[47]. Animal models support the hypothesis of vulnerability in spatial learning suggestive of hippocampal impairment; alcohol-exposed rats are impaired at the Morris water maze, T-mazes, and radial arm mazes[48]. There is evidence that children and adolescents exposed to alcohol prenatally have similar visuospatial memory deficits. Comparable deficits in Morris water maze learning is evident[49], and Uecker and Nadel[50] found particular problems with object location and only subtle weaknesses in object memory, although others have found spatial deficits to be confounded with impaired verbal memory[51]. Indeed, verbal memory may not be spared and deficits are reported[25].

Memory impairment has a variety of forms, and encompasses recall omission, intrusions, perseverations, and poor recognition discrimination[46]. Impairments in recall are more consistently reported than deficits in recognition[52] and impairments may affect explicit tasks more so than implicit[53]. Even in infants, recognition memory may be relatively spared[54]. Furthermore, verbal learning is impaired, but there does appear to be normal long-term retention of previously learned visual and verbal material. For example, difficulty learning a word list is not related to difficulty in retaining the list once learned[55]. Priming also seems to be effective and there are typical benefits of previous exposure in FASDs relative to controls[53]. Memory impairment in FASDs is complex; both visual and verbal recall is affected, but recognition, long-term retention, implicit memory, and specific subtypes of memory (e.g., facial memory[50]) may be spared.

## Socioemotional Functioning

Socioemotional functioning, defined as the interplay between affective processing and social behavior, is not well understood in FASDs. As typical development of language and executive functioning may be critical for the development of social cognition, impairments may have a cascading impact on socioemotional development[35,56]. Indeed, degree of cognitive dysfunction reliably predicts behavior problems in FASDs[41]. The problems in socioemotional behavior also exceed what would be expected based on IQ alone and seem to reflect an arrest, rather than a delay, in function[57].

Key problems in atypical attachment and difficulty regulating state[58] begin in early infancy with irritability, disturbed sleep patterns, and feeding problems. In early life, the children may present as talkative, rambling, and overly inquisitive, therefore overshadowing behavior problems[29]. At approximately school age, children with FASDs show an arrest in socioemotional development[59], with problems typically presenting around 6 years of age[60]. Steinhausen[61] reports that in childhood, severe deficits in the social domain are seen, highlighted by characteristic reports of impulsivity, aggression, and mood lability. More subtle deficits in the ability to infer from another's perspective (theory of mind), in the reading of social cues, and in social withdrawal negatively impacts social interaction. Parents report that the children are highly prone to hyperactivity, disruptivity, impulsivity, and delinquency[62,63], and teachers report less social competency and greater aggression in the classroom[64]. The negative behavior is accompanied by a lack of guilt, an inability to learn from past mistakes, and deficits in moral judgments as they pertain to relationships with others[19,65]. Parent ratings suggest that the most salient deficit may be improper socialization as it relates to responding to social cues, considering consequences of actions, and interpersonal skills[66].

The socioemotional defects evident in infancy do not attenuate over time, but rather increase with age[57] and may actually set the stage for disrupted socioemotional function across the lifespan[58]. Similarly, maladaptive social behaviors may have a more detrimental impact with age, as behaviors that seem cute or endearing at a young age become increasingly inappropriate. For instance, excessive friendliness and affection[67] raise concerns about safety and appropriate social interaction[29]. The long-term consequences of socioemotional impairment are distressing.

## Psychopathology

Socioemotional deficits may precede the onset of psychopathology or other secondary disabilities that include mental health disorders (90%), drug and alcohol abuse (50%), trouble with the law (50%), trouble at school (60%), and employment problems (70%), to name a few[29], often putting individuals at risk for developing psychopathology. In a recent attempt to address this issue, Fryer et al.[68] examined psychopathological conditions (secondary disabilities) in a community sample of children with FASDs. Results revealed that 97% of the FASDs sample met criteria for a DSM-IV axis I disorder. Compared to controls, children with FASDs were more likely to meet criteria for ADHD, depressive disorders, oppositional defiant disorder (ODD), conduct disorder (CD), and specific phobia. Perhaps most notable from their findings is that there were substantially more comorbid diagnoses in the alcohol-exposed group (71%) than the control group, particularly among the disruptive disorders. This finding is especially important as many of the social and emotional problems reported by parents and teachers of children with FASDs are disruptive in nature[19,35].

One aspect of psychopathology that has received little attention in adolescents with FASDs is moral maturity. Using Kohlberg's stages of moral development, Schonfeld and colleagues[56] were the first to examine this issue; compared to controls, children with FASDs were focused on reducing negative consequences on the self (stage 2), whereas controls demonstrated concern for others and social norms (stage 3). Additionally, children with FASDs were found to have poor social judgment, trouble learning from experience, failure to consider the consequences of their actions, and demonstrated difficulty communicating in social contexts. Most notable from this study is the finding that underdeveloped levels of moral maturity in children with FASDs were related to elevated levels of delinquency, particularly stealing and conduct problems. Interestingly, similar results were found by Nash et al.[19], using individual items from the Child Behaviour Checklist.

Recently, efforts have been made to elucidate the early manifestations and specific underpinnings of the social and emotional disturbances seen in FASDs. However, what remains striking is that the majority of research efforts continue to focus heavily on the cognitive deficits, whereas secondary disabilities and their precursors remain largely ignored in individuals with FASDs.

## CONCLUSIONS

Research examining the FASDs profile has evolved tremendously since FAS was first introduced into the medical literature in the 1960s. It is clear that early intervention and a positive rearing environment provides the best chance of circumventing the devastating secondary disabilities for individuals with FASDs. However, research also indicates that we are far from understanding the full spectrum of strengths and weaknesses that typify individuals with FASDs, thus impeding our ability to identify a specific neurobehavioral phenotype. Identifying the phenotype is further complicated by the fact that many children with FASDs also share profiles or comorbid diagnoses of children with other clinical conditions, such as ADHD, ODD, and CD. Therefore, it will be especially important for future research to delineate the FASDs profile from these other childhood disorders. Until then, it will be difficult to develop interventions that specifically target FASDs. On a societal level, with the cost estimated to be up to 1.4 million dollars in intervention across the lifespan of an individual with FASDs[4], FASDs remain a critical public health concern and can only be alleviated with early intervention initiatives. In both economic and individual terms, the costs of the impact of the disorder on the families of individuals with FASDs are immeasurable. Consequently, in so far as it is possible, the primary goal of research efforts should be to provide hope for individuals with FASDs and their families.
